# Free Vibration Analysis of Patch Repaired Plates with a Through Crack by *p*-Convergent Layerwise Element

**DOI:** 10.1155/2014/427879

**Published:** 2014-08-17

**Authors:** Jae S. Ahn, Seung H. Yang, Kwang S. Woo

**Affiliations:** Department of Civil Engineering, Yeungnam University, 280 Daehak-Ro, Gyeongsan, Gyeongbuk 712-749, Republic of Korea

## Abstract

The high-order layerwise element models have been used for damaged plates and shells in the presence of singularities such as crack, cutout, and delamination. In this study, the extension of a proposed finite element model has been tested for free vibration analysis of composite laminated systems. For the elements, three-dimensional displacement fields can be captured by layer-by-layer representation. For the elements, higher-order shape functions are derived by combination of one- and two-dimensional shape functions based on higher-order Lobatto shape functions, not using pure higher-order three-dimensional shape functions. The present model can relieve difficulty of aspect ratios in modeling very thin thickness of bonding layer. For verification of the model, natural frequencies and corresponding mode shapes are calculated and then compared with reference values for uncracked and cracked plates. Also, the vibration characteristics of one-sided patch repaired plates with a through internal crack are investigated with respect to variation of crack length, size and thickness of patch, and shear modulus of adhesive, respectively.

## 1. Introduction

For enhancement of service life in structures with local damage or defect, adhesively bonded technology has widespread applications in aircraft, ship, and other structures due to its light weight and efficient load transfer characteristics. Composite patches, especially, have shown to be a highly cost effective method for extending the service life and maintaining high structural efficiency [1–3]. To investigate the behavior of damaged structures repaired by composite patches, stress intensity factors obtained from stress analysis have often been considered, which are reduced by the presence of the patches. For the stress analysis, some authors have addressed various analytical, numerical, and experimental aspects. As the analytical solutions [4–6] could not effectively handle the complexities of real-life patch-repair problems, the emphasis has been on experimental and numerical methods. In the case of numerical methods, most authors [7–9] have relied upon conventional finite element analysis based on *h*-refined mesh design, utilizing hexahedral elements for three-dimensional modeling and plate elements for two-dimensional modeling. In three-dimensional models, discretization of the extremely thin adhesive layer with hexahedral elements with acceptable aspect ratios led to models with unacceptable large number of elements. Ahn and Basu [10] proposed a mixed-model approach to analyze cracked metal plates with patch repair, which shows a strong robustness of the element with respect to very large aspect ratios of 1:200 for extremely thin adhesive modeling. Some other authors [11, 12] have focused their attention on the optimal design of the bonded patches by finite element models.

Although static analyses of patch repaired plates with a crack have intensively been performed to determine stress intensity factors, investigations on their free vibrations of patch repaired plates with a crack are rather little. Researches on vibration analysis of some plates including through internal cracks have often been implemented. Stahl and Keer [13] reported vibration phenomenon of cracked rectangular plates with simple supports whose analysis is based on a dual series equation. Solecki [14] studied vibration of rectangular plates with a crack parallel to one edge using a finite Fourier series transformation in conjunction with the generalized Green-Gauss theorem. Liew et al. [15] used domain decomposition method in determining frequencies of cracked plates. Wu and Shih [16] studied dynamic instability of rectangular plates with an edge crack. Bachene et al. [17] used extended finite element method based on Mindlin plate theory for vibration analysis of cracked plates. Recently, Ritz method considering shear deformation was applied for determining frequencies and nodal patterns of thick, cracked rectangular plates [18]. To the author's knowledge, it is nearly impossible to find the published literatures about the free vibration analysis of patch repaired plates with a through internal crack. Natural frequency is one of the significant characteristics in engineering applications and dynamic responses of the cracked structure that may change after patching. Hence, it is necessary to understand the variation of the natural frequencies of the patched and unpatched cracked structure for effective patching design.

Meanwhile, the quest for the robust finite elements for a wide class of practical problems involving stress singularities has triggered researchers to develop higher-order finite elements. Mathematical justification showing the advantages of higher-order approximations of the field variables has been reported [19] regarding high accuracy, high convergence rate, coarse mesh, and improved performance in handling stress singularity problems. As previously worked, fracture analysis using the *p*-convergent layerwise elements [20] based on hierarchical shape functions was implemented in which three-dimensional displacement fields can be captured by layer-by-layer representation. For the elements, higher-order shape functions are derived by combination of one- and two-dimensional shape functions based on Lobatto shape functions, not using pure higher-order three-dimensional shape functions. Then, stress intensity factors of cracked plates with a patch repair [10, 21] were obtained by the *p*-convergent layerwise elements. In this paper, the proposed elements are applied to the free vibration analysis of cracked plates with a patch repair. For verification of the proposed elements, at first, natural frequencies and the corresponding mode shapes are compared with reference values for uncracked and cracked plates. Then, vibration characteristics of one-sided patch repaired plates are investigated on natural frequencies in terms of crack length, size and thickness of patch, and shear modulus of adhesive.

## 2. *p*-Convergent Layerwise Element Model

### 2.1. Displacement Fields

In this approach [20] to three-dimensional modeling of patch repaired systems, each layup is treated discretely with deformation of a point in the layup in terms of three-displacement components defined for each layer separately. Displacement field at bottom and top surfaces within a layer is approximated by two-dimensional shape functions. Then the two surfaces are connected by interpolating technique using one-dimensional shape functions which are first-order or more variations across thickness. The one-dimensional hierarchical shape functions can be classified into two groups as nodal modes (*F*) and nodeless modes (*B*). For two-dimensional hierarchical shape functions, three-mode groups belong to the quadrilateral element such as nodal modes (*N*) and nodeless modes (*M*) including side and internal modes. Generally, the nodal modes have physical meaning, while the nodeless modes with respect to the increase of order of the Lobatto shape function do not have physical meaning but improve accuracy of analysis. The displacement field {Φ} consisting of three components (*U*, *V*, and *W*) at a point (*x*, *y*, *z*) can be written as

**Figure 1:**
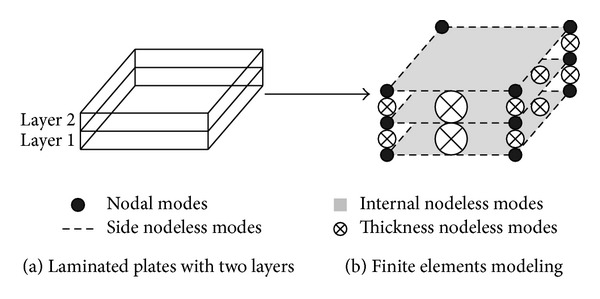
Modeling scheme of laminate plates with two layers using *p*-convergent layerwise element.

(1) U=NiFjuij+NiBsais+MkFjbkj+MkBscks,i=1,2,3,4;  j=1,2;V=NiFjvij+NiBsdis+MkFjekj+MkBsfks,k=1,2,…,p(p+3)2−1;W=NiFjwij+NiBsgis+MkFjhkj+MkBsqks,s=1,2,…,p′−1,

where for the sake of brevity the Einstein summation convention has been introduced for a repeated index. *u*_*i*_^*j*^, *v*_*i*_^*j*^, and *w*_*i*_^*j*^ are the nodal variables, and *a*_*i*_^*s*^, *b*_*k*_^*j*^, *c*_*k*_^*s*^, *d*_*i*_^*s*^, *e*_*k*_^*j*^, *f*_*k*_^*s*^, *g*_*i*_^*s*^, *h*_*k*_^*j*^, and *q*_*k*_^*s*^ are nodeless variables. The number of the nodeless variables depends on order of the approximation functions, *p* and *p*′ (≥2), which are independent of each other.  Figure 1 depicts the modeling scheme with present elements for a laminated system with two layers. If there are no gaps and empty spaces between interfaces of layers, compatibility conditions can be applied at the layer interfaces. Each layer has eight nodal modes. Also, it takes side, internal, and thickness nodeless modes of which numbers depend on the order of the approximation functions adopted, *p* and *p*′.

### 2.2. Shape Functions

For the functions stated above, at first, one-dimensional shape functions with higher-order degrees are adopted from the Lobatto shape functions [22] defined within the space (−1 ≤ *x* ≤ 1) that are given by

(2) $$\begin{array}{c} F_{1}\left(x\right)=\frac{1-x}{2},\;\;F_{2}\left(x\right)=\frac{1+x}{2},\\ B_{s}\left(x\right)=\frac{1}{||L_{s}||}\overset{x}{\underset{-1}{\int }}L_{s}\left(\xi \right)d\xi ,\;\;s\geq p^{\prime }-1, \end{array}$$

where

(3) $$\begin{array}{c} ||L_{s}||=\sqrt{\frac{2}{2s+1}}. \end{array}$$

The higher-order Legendre polynomials, *L*_*s*_, can be defined by differential relations as follows:

(4) $$\begin{array}{c} L_{s}\left(x\right)=\frac{1}{2^{s}s!}\frac{d^{s}}{dx^{s}}{\left(x^{2}-1\right)}^{s},\;\text{for}\;\;s=0,1,2,\ldots . \end{array}$$

Their orthogonal relationship is exactly specified by

(5) $$\begin{array}{c} \overset{x}{\underset{-1}{\int }}L_{i}\left(x\right)L_{j}\left(x\right)dx=\{\begin{array}{cc} \frac{2}{2i+1} & \text{for}\;\;i=j\\ 0 & \text{otherwise}. \end{array} \end{array}$$

The one-dimensional Lobatto shape functions derived from the higher-order integrals of Legendre polynomials play an essential role in the design of two-dimensional hierarchical shape functions for this discrete layer model. The two-dimensional shape functions associated with the values of nodes are given by

(6) $$\begin{array}{c} X_{i,j}=F_{i}\left(x\right)F_{j}\left(y\right),\;i,j=1,2, \end{array}$$

where

(7) $$\begin{array}{c} N_{1}=X_{1,1},\;\;N_{2}=X_{2,1},\;\;N_{3}=X_{2,2},\;\;N_{4}=X_{1,2}. \end{array}$$

In any *p*-levels (*p* ≥ 2), two-dimensional shape functions associated with nodeless variables are as follows:

(8) $$\begin{array}{c} M_{0.5i(i+3)+\alpha }=B_{i}\left(x\right)F_{1}\left(y\right),\\ M_{0.5(i+1)(i+2)+\alpha }=F_{2}\left(x\right)B_{i}\left(y\right),\\ M_{0.5(i^{2}+3i+4)+\alpha }=B_{i}\left(x\right)F_{2}\left(y\right),\\ M_{0.5(i^{2}+3i+6)+\alpha }=F_{1}\left(x\right)B_{i}\left(y\right),\\ i=1,2,\ldots ,p-1;\;\;\alpha =\{\begin{array}{cc} -1 & \text{in}\;\;i=1\\ 0 & \text{otherwise}. \end{array} \end{array}$$

For *p* ≥ 4, the additional shape functions of nodeless variables are obtained by

(9) M0.5(j2+j+1)+i=Bi(x)Bj−i−2(y); i=1,2,…,j−3for  j=4,5,…,p.

### 2.3. Strain Fields

For a typical layer, *l*, stress-strain relationships, which are based on three-dimensional elasticity theory, are linear as follows:

(10) $$\begin{array}{c} {\left\{\sigma_{x,y,z}\right\}}_{6\times 1}^{l}={\left[D\right]}_{6\times 6}^{l}{\left\{\varepsilon_{x,y,z}\right\}}_{6\times 1}^{l}. \end{array}$$

Here, [*D*] is a general elasticity matrix of orthotropic materials and strain matrix is given by

(11) {εx,y,z}=⌊∂U∂x∂V∂y∂W∂z∂U∂y+∂V∂x∂U∂z+∂W∂x∂V∂z+∂W∂y⌋T.

### 2.4. Equation of Motion

Lagrange equations for most structural mechanics problems may be derived from consideration of Hamilton's principle that is well known in the text. For free vibration problems without damping, the governing equation of motion requires the functional to satisfy the condition as follows:

(12) $$\begin{array}{c} \overset{}{\underset{t}{\int }}\delta \left(T-U\right)dt=0, \end{array}$$

where *T* is total kinetic energy, *U* is potential energy including both strain energy and potential energy of any conservative external forces, and *δ* is a variation taken during the indicated time interval *t*. The displacement fields {Φ} defined in (1) can be written by the following general form:

(13) $$\begin{array}{c} \left\{\Phi \right\}=\left[H\right]\left\{d\right\}, \end{array}$$

where all nodal and nodeless variables are included in the matrix {*d*} and the matrix [*H*] indicates hierarchical shape functions defined in (7)–(9). First-order derivative of the displacement fields with respect to time is given by

(14) $$\begin{array}{c} \left\{\dot{\Phi }\right\}=\left[H\right]\left\{\dot{d}\right\}. \end{array}$$

Then, the kinetic energy *T* can be written by

(15) $$\begin{array}{c} T=\frac{1}{2}\overset{}{\underset{V}{\int }}\rho {\left\{\dot{\Phi }\right\}}^{T}\left\{\dot{\Phi }\right\}dV. \end{array}$$

Also, from the strain vector {*ε*} and the stress vector {*σ*} defined in (10) and (11), the potential energy *U* can be written as

(16) $$\begin{array}{c} U=\frac{1}{2}\overset{}{\underset{V}{\int }}{\left\{\varepsilon \right\}}^{T}\left\{\sigma \right\}dV. \end{array}$$

Thus the energy functional expressed in matrix form can be obtained as follows:

(17) ∫tδ(12∫Vρ{d˙}T[H]T[H]{d˙}dV−12∫V{d}T[B]T[D][B]{d}dV)dt=0,

where [*B*] is the strain-displacement matrix with respect to layer reference axes, [*D*] is an elasticity matrix with an orthotropic material. The total kinetic energy, the first term of (17), is a functional with respect to displacements and velocities, while the potential energy is a functional with respect to only displacements. The velocity-related term in (17) is integrated by parts and then the minimization of energy functional is applied. Then by differentiating (17) with respect to time, the final equation of motion for free vibration problems for undamped system can be expressed in matrix form as

(18) $$\begin{array}{c} \left[M\right]\left\{\ddot{d}\right\}+\left[K\right]\left\{d\right\}=0, \end{array}$$

where

(19) $$\begin{array}{c} \left[M\right]=\overset{}{\underset{V}{\int }}\rho {\left[H\right]}^{T}\left[H\right]dV,\\ \left[K\right]=\overset{}{\underset{V}{\int }}{\left[B\right]}^{T}\left[D\right]\left[B\right]dV. \end{array}$$

Natural vibration is nothing but the periodic motion with any natural circular frequencies *w*. By assuming the proper periodic motion, (20) can be obtained:

(20) $$\begin{array}{c} \left[\left[K\right]-w^{2}\left[M\right]\right]\left\{d\right\}=0. \end{array}$$

When (20) has a nontrivial solution, characteristic matrix of {*d*} should be singular matrix to satisfy the condition as follows:

(21) $$\begin{array}{c} \left|\left[K\right]-w^{2}\left[M\right]\right|=0. \end{array}$$

Using a commercial package like MATLAB, characteristic equation (21) to find natural circular frequencies and the corresponding mode shapes can be solved.

## 3. Numerical Examples

### 3.1. Cracked Square Plates

**Figure 2:**
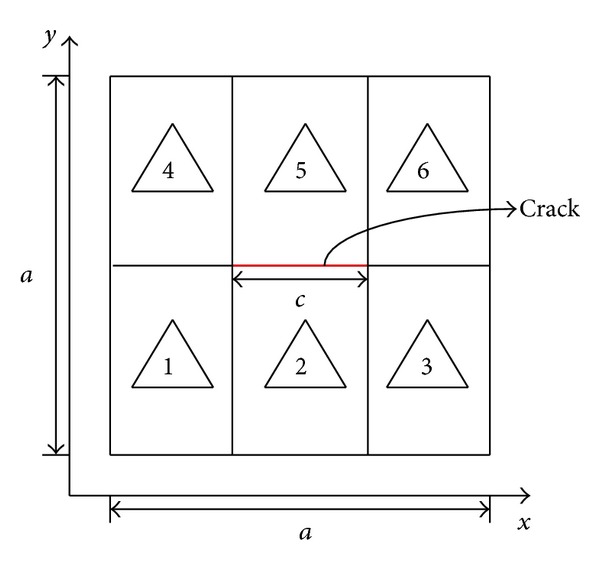
Modeling of cracked plates by the *p*-convergent layerwise elements.

**Figure 3:**
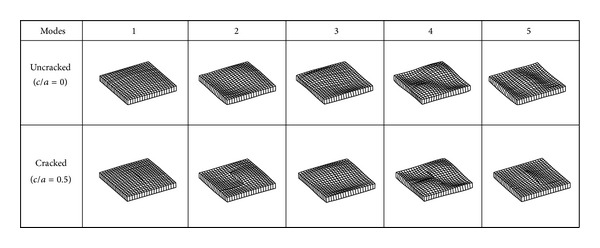
Mode shapes of uncracked and cracked plates.

The free vibration of simply supported square plates with a center crack is considered when *a*/*t* ratio is fixed as 10, where *a* and *t* represent the side and thickness of square plate, respectively. The plates are discretized into 3 × 2 elements like in Figure 2. Based on convergence tests, the orders of polynomial approximation are kept to 6 and 3 in plane and along thickness, respectively. To facilitate comparison of natural circular frequencies (*ω*), the nondimensional frequency parameter *λ* is considered as

(22) $$\begin{array}{c} \lambda =\frac{\omega a^{2}}{\pi^{2}t}\sqrt{\frac{12\rho \left(1-\nu^{2}\right)}{E}}, \end{array}$$

where *ρ* is material density of the plates, *E* is Young's modulus, and *ν* is Poisson's ratio. The first five nondimensional frequency parameters are presented in Table 1 for different crack lengths (*c*/*a* = 0.1, 0.2, 0.3, 0.4, and 0.5) where *c* is the crack length of plates and then are compared with reference values [18]. It should be pointed out that the present results are in good agreement with the reference values within the relative error of ±2% for all cases. It is true that frequencies are reduced with the increase of crack. The fundamental frequency of the cracked plate with *c*/*a* = 0.5 is reduced up to 12% as compared with that of the uncracked plate with *c*/*a* = 0. Also, it is seen that the reductions of frequencies are much larger for the second and the fifth modes than for the other modes. The frequencies may, respectively, be reduced by about 19% for the second mode and 17% for the fifth mode, while reductions of the third and fourth modes are within 5%. It means that first, second, and fifth mode shapes of first five modes are more dependent on crack size than the other mode shapes.  Figure 3 shows the first five vibration mode shapes of uncracked and cracked plates to present the influence of a crack. It is observed how the cracks split the plates into two parts according to mode shapes.

### 3.2. Cracked Square Plates with a Patch Repair

**Figure 4:**
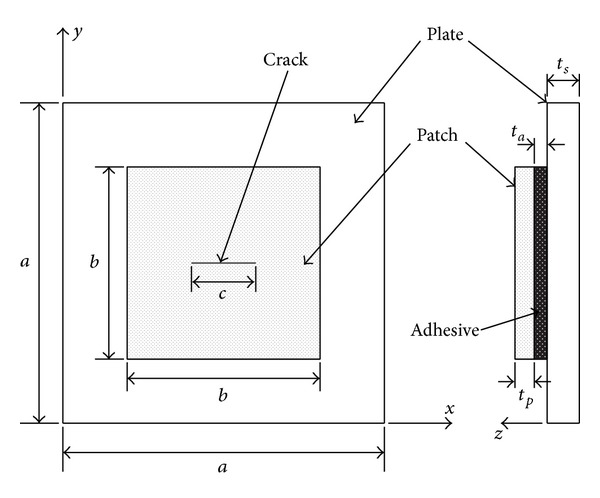
Geometry of center-cracked plates with externally bonded repairs.

**Figure 5:**
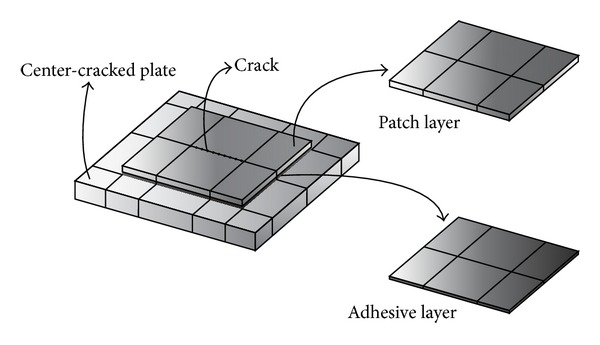
Modeling of cracked plates with a single-sided patch repair using present elements.

A repair method using perfectly bonded composite patch covering a structural defect can be used to enhance the service. In this study, the center-cracked steel plates with a single-sided patch repair are considered as shown in Figure 4. To obtain fundamental frequencies of the patched problems and to investigate effect of some parameters, the present model is applied. The plates have the following dimensions: length *a* = 300 mm, thickness *t*_*s*_ = 30 mm, patching length *b* = 180 mm, *t*_*p*_ = 10 mm, and *t*_*a*_ = 1.5 mm, respectively. For patching material, composite material with combination of boron and epoxy is adopted. The elastic properties of the steel, film adhesive, and boron/epoxy are given in Table 2. If there are no additional conditions of geometry and materials in specific cases, those values aforementioned are chosen. For finite element meshing work, the steel plates are discretized into 5 × 4 elements, and 3 × 2 mesh design is used for patch and adhesive layers as shown in Figure 5. Like the pervious problem, the orders of polynomial approximation are kept to 6 and 3 in plane and along thickness, respectively, through the convergence tests.

**Figure 6:**
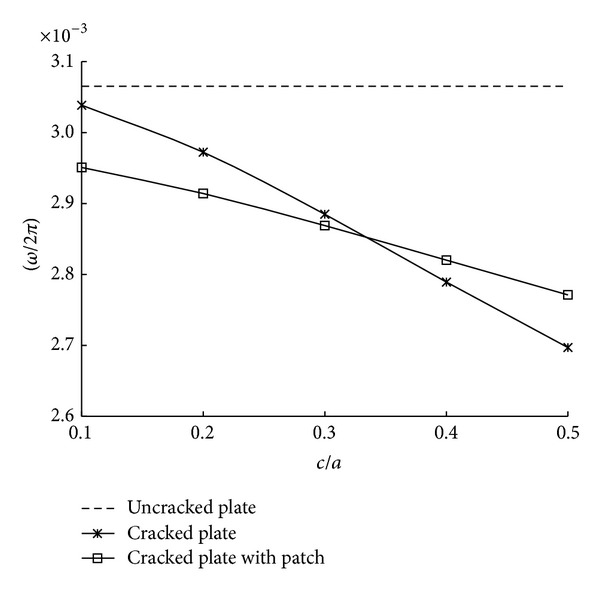
Variation of natural frequencies with crack size.

At first, Figure 6 shows the variation of natural frequencies with crack size for cracked plates with and without patch, respectively, in which the values of the cracked plates are compared with the value of an uncracked plate. It is seen from the results that the natural frequencies of all cracked plates with and without patch are smaller than that of the uncracked plate. The natural frequencies of the patched plates are somewhat larger than those of the cracked plates without patch below *c*/*a* = 0.3. When crack size is small, total mass has more influence on natural frequencies than stiffness. The figure also shows that decreasing ratio of the values in the patched plates is smaller than that of the unpatched plates with crack. It is noted that the relatively large stiffness effect by patching can lower the decreasing ratio of natural frequencies.

**Figure 7:**
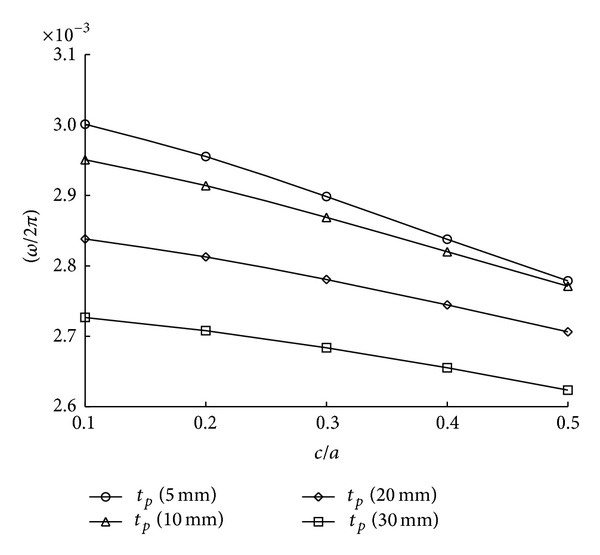
Variation of natural frequencies with different patch thickness.

**Figure 8:**
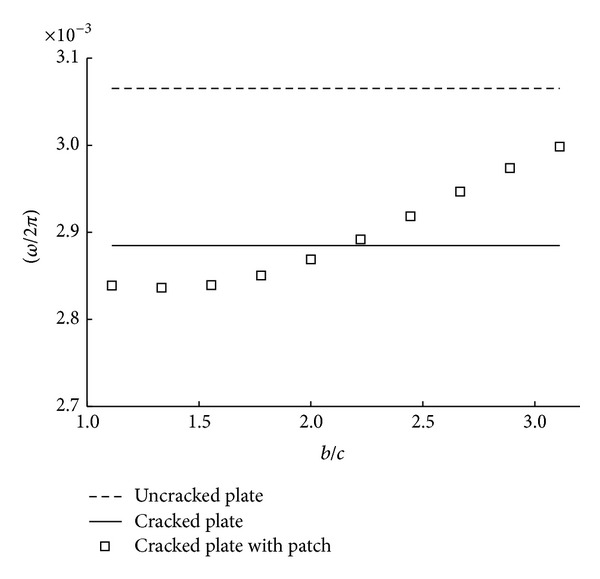
Variation of natural frequencies with patch length.

Figure 7 illustrates the variation of natural frequencies according to different patch thickness varying from *t*_*p*_ = 5 mm to *t*_*p*_ = 30 mm as a central crack propagates. In this case, the adhesive thickness *t*_*a*_ is fixed as 0.75 mm and other dimensions and material properties are exactly the same as the values mentioned in Table 2. As we are aware of it, the patch repair generally reduces stress intensity factors significantly up to a certain level of patch thickness since the stiffness of cracked plates enforced by patching effect is increased. However, it is noted from Figure 7 that growth of patch thickness decreases the natural frequencies of patch repaired plates when the plates have the same crack length. This is why the mass increment is more dominant to natural frequency than to the increase of stiffness.  Figure 8 shows variation of natural frequencies with patch length *b* from 100 mm to 280 mm where crack length *c* is fixed to 90 mm. When patch length is smaller than double length of crack size, it is seen that patched plates have smaller frequencies than cracked plates without patch. The phenomenon occurs because stiffness intension by patching is smaller than mass increase by adding patching materials, boron/epoxy and adhesive. When patching effect is enough, natural frequencies are close to those of the uncracked plates. It can be told that variation of patch length has more positive influence than that of patch thickness as illustrated by the results of Figures 7 and 8.

**Figure 9:**
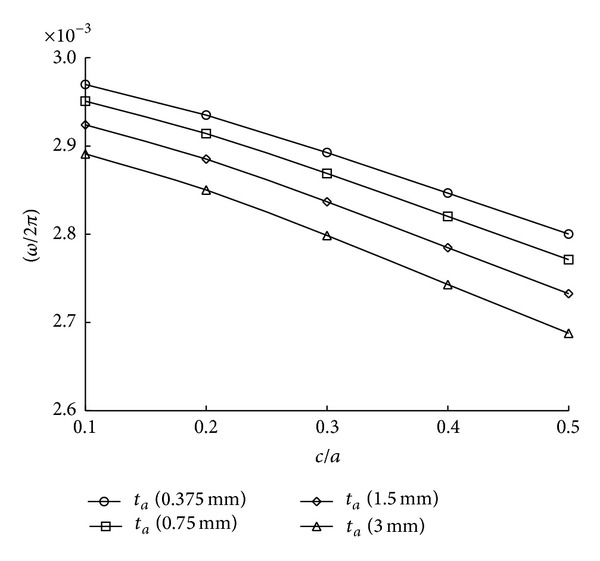
Variation of natural frequencies with adhesive thickness.

**Figure 10:**
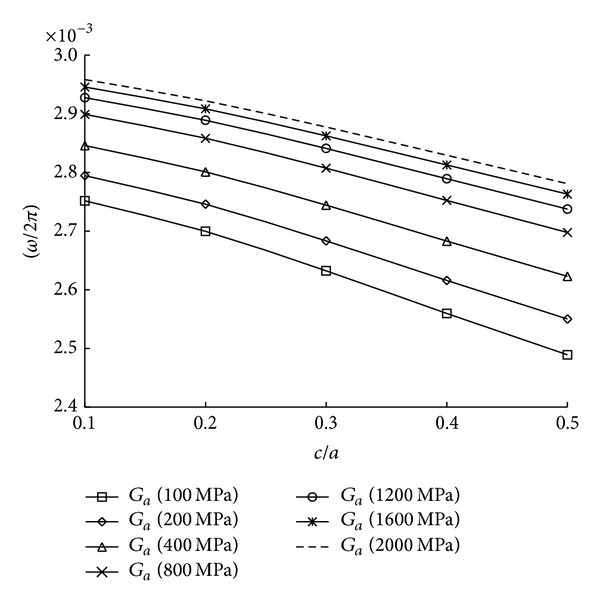
Variation of natural frequencies with different adhesive shear modulus.

Next, influence of crack length and thickness of adhesive layer is given in Figure 9. It can be told that the increase of the adhesive thickness decreases the fundamental frequencies. It is why the increase of the adhesive thickness causes a mass increment. The natural frequencies may be slightly reduced to approximately 2.7% for *c*/*a* = 0.1 and 4.2% for *c*/*a* = 0.5 although octuple increase of the adhesive thickness is given from *t*_*a*_ = 0.375 mm to *t*_*a*_ = 3 mm. Therefore, in practical cases of patching problems, effect of adhesive thickness may be negligible for frequency values, since variation of adhesive thickness is very small.  Figure 10 presents influence of shear module of adhesives depending on variation of crack length. The increase in the natural frequencies due to single patching effect can be approximately between 7.5% for *c*/*a* = 0.1 and 11.7% for *c*/*a* = 0.5 when the adhesive shear modulus is varied from 100 MPa to 2000 MPa. It is also noted that the natural frequencies decrease as the crack length is increased. From Figures 9 and 10, it is observed that the adhesive shear modulus has more significant effect on variation of the natural frequencies as compared with the adhesive thickness.

## 4. Conclusions

The aim of this study is to show the efficiency of the proposed *p*-convergent layerwise model for the free vibration analysis of cracked square plates without and with patch. Also, this study is extended to single patching effect of cracked plates. The obtained results deduce the following conclusions.

1. Since the proposed *p*-convergent layerwise model tolerates the large aspect ratio, the number of meshes can be drastically reduced as compared with the conventional solid element, especially in the case of considering very thin adhesive and patch.
2. Frequency of each mode is reduced with increase of the crack length because stiffness of cracked plates decreases. Particularly, it can be told that frequencies of first, second, and fifth modes are largely decreased more than those of third and fourth modes.
3. The patching effect can help decreasing ratios of natural frequencies reduce as crack size increases.
4. Increase of patch length has more positive effect than increase of patch thickness in order to be close to the natural frequencies of original plates prior to damage.
5. It is observed that the shear modulus of adhesive has more influence on the natural frequency as compared with the adhesive thickness.
6. From these results, in future it is necessary to investigate the suitable size and thickness of patch before the design of optimal patching systems.

## Figures and Tables

**Table 1:** Comparison of nondimensional frequencies with respect to variation of crack lengths.

*c*/*a*	Model	Mode				
		1	2	3	4	5
0	Huang et al. (2011) [18]	1.9312	4.6050	4.6050	7.0641	8.6052
	Present	1.9346	4.6242	4.6242	7.1070	8.6683

0.1	Huang et al. (2011) [18]	1.9109	4.5949	4.6050	7.0479	8.4299
	Present	1.9177	4.6168	4.6240	7.0943	8.5153

0.2	Huang et al. (2011) [18]	1.8673	4.5432	4.6010	7.0195	8.0753
	Present	1.8758	4.5729	4.6208	7.0677	8.1654

0.3	Huang et al. (2011) [18]	1.8116	4.4034	4.5878	6.9820	7.7004
	Present	1.8206	4.4473	4.6092	7.0311	7.7802

0.4	Huang et al. (2011) [18]	1.7518	4.1187	4.5615	6.9121	7.3833
	Present	1.7604	4.1832	4.5839	6.9666	7.4516

0.5	Huang et al. (2011) [18]	1.6941	3.6911	4.5210	6.7693	7.1431
	Present	1.7021	3.7685	4.5433	6.8355	7.2001

**Table 2:** Material properties.

Material	*E*_1_ (GPa)	*E*_2_, *E*_3_ (GPa)	*G*_12_, *G*_13_ (GPa)	*G*_23_ (GPa)	***ν***_**12**_, ***ν***_**13**_, ***ν***_**23**_	*ρ* (kg/mm^3^)
Steel	200	200	76.9	76.9	0.3	2.7 × 10^−6^
Film adhesive	3.068	3.068	1.138	1.138	0.35	0.33 × 10^−6^
Boron/epoxy	223.4	24.13	8.481	5.275	0.23	2.1 × 10^−6^
